# Assessment of alcohol problems using AUDIT in a prison setting: more than an 'aye or no' question

**DOI:** 10.1186/1471-2458-11-865

**Published:** 2011-11-14

**Authors:** Susan MacAskill, Tessa Parkes, Oona Brooks, Lesley Graham, Andrew McAuley, Abraham Brown

**Affiliations:** 1 Institute for Social Marketing, University of Stirling, Stirling, UK; 2 School of Nursing, Midwifery and Health, University of Stirling, Stirling, UK; 3 School of Health & Social Sciences, University of Abertay, Dundee, UK; 4 Information Services Division, National Services Scotland, Gyle Square, 1 Gyle Crescent, Edinburgh, UK; 5 Public Health Science Directorate, NHS Health Scotland, Glasgow, UK

## Abstract

**Background:**

Alcohol problems are a major UK and international public health issue. The prevalence of alcohol problems is markedly higher among prisoners than the general population. However, studies suggest alcohol problems among prisoners are under-detected, under-recorded and under-treated. Identifying offenders with alcohol problems is fundamental to providing high quality healthcare. This paper reports use of the AUDIT screening tool to assess alcohol problems among prisoners.

**Methods:**

Universal screening was undertaken over ten weeks with all entrants to one male Scottish prison using the AUDIT standardised screening tool and supplementary contextual questions. The questionnaire was administered by trained prison officers during routine admission procedures. Overall 259 anonymised completed questionnaires were analysed.

**Results:**

AUDIT scores showed a high prevalence of alcohol problems with 73% of prisoner scores indicating an alcohol use disorder (8+), including 36% having scores indicating 'possible dependence' (20-40).

AUDIT scores indicating 'possible dependence' were most apparent among 18-24 and 40-64 year-olds (40% and 56% respectively). However, individual questions showed important differences, with younger drinkers less likely to demonstrate habitual and addictive behaviours than the older age group. Disparity between high levels of harmful/hazardous/dependent drinking and low levels of 'treatment' emerged (only 27% of prisoners with scores indicating 'possible dependence' reported being 'in treatment').

Self-reported associations between drinking alcohol and the index crime were identified among two-fifths of respondents, rising to half of those reporting violent crimes.

**Conclusions:**

To our knowledge, this is the first study to identify differing behaviours and needs among prisoners with high AUDIT score ranges, through additional analysis of individual questions. The study has identified high prevalence of alcohol use, varied problem behaviours, and links across drinking, crime and recidivism, supporting the argument for more extensive provision of alcohol-focused interventions in prisons. These should be carefully targeted based on initial screening and assessment, responsive, and include care pathways linking prisoners to community services. Finally, findings confirm the value and feasibility of routine use of the AUDIT screening tool in prison settings, to considerably enhance practice in the detection and understanding of alcohol problems, improving on current more limited questioning (e.g. 'yes or no' questions).

## Background

Alcohol problems are a major public health issue in the UK. The consequences affect individuals, their families, the health and emergency services and wider society. The strong association between alcohol consumption and an individual's risk of being either a perpetrator or victim of violent crime has been identified internationally [1]. The extent of alcohol problems in UK and Scottish offender populations is also being increasingly recognised [2-4].

In Scotland, alcohol is known to be closely associated with domestic abuse [5] and is a risk factor in both the social patterning of assault [6] and facial injury [7]. The Scottish Crime and Justice Survey 2009/10 [8] reports that in 62% of violent crime the victims perceived offenders to be under the influence of alcohol (the equivalent figure for drugs was 26%). Where known, alcohol is also a factor in 69% of homicide cases [9], while 70% of assaults in Scottish Accident and Emergency departments are likely to be alcohol-related [10], the majority of these involving young men. In addition, alcohol treatment was a condition of 10% of probation orders (community sentences) in 2008/09 [11]. Overall costs of alcohol misuse in Scotland are estimated to be £3.6 billion (based on mid-point estimates) with alcohol-related crime accounting for over £700 million [12].

The prevalence of alcohol problems is markedly higher in the Scottish prison population compared to the general population, at all ages and for both women and men, as shown in comparative analysis conducted as a separate part of this study [13]. Among male and female prisoners, 44% and 48% respectively responded positively to two or more questions from CAGE, a four question screening tool where two or more positive responses indicate problematic alcohol use. This compares with 13% and 9% males and females in the general population in response to equivalent questions. Among 16-24 year-olds, the prevalence was more than two-and-a-half times greater among men in prison, and three-and-a-half times greater among women. Among women in prison aged 45-54 years, 54% were likely to have an alcohol problem, more than five times the equivalent general female population figure [13].

It is important to put alcohol-related offending into a broader social and economic context. Prisoners in Scotland are predominantly young men from disadvantaged backgrounds, many of whom have substance misuse problems [14]. The Scottish Health Survey 2009 [15] showed that young men were the group most likely to drink to excess and that men living in the most deprived areas of the country are likely to drink the most. According to Richardson and Budd [16], binge drinkers are those most likely to offend. Alcohol-related problems in offenders also co-exist with drug-related and mental health problems, as well as a range of other health and social problems, resulting in a complex picture of individual need [2,14,17-19]. A health care needs assessment carried out in the Scottish Prison Service (SPS) in 2007 identified key areas for service development in SPS healthcare to address some of these complex and interconnecting problems [14]. These key areas included more health-related services for those on short term sentences and on remand (i.e. in custody, pending trial) and the strengthening of links with community services and agencies, both on the way into prison and on liberation. More specifically the assessment recommended formal screening for alcohol problems on admission and the piloting and evaluation of brief interventions for those with mild to moderate alcohol problems staying for short periods. It also identified the need for better integration between healthcare and substance misuse specialist services, both within the prison estate and on the way into and out of prison.

Identifying individuals with alcohol problems is fundamental to providing high quality interventions tailored to individual needs in prison settings. It is also a necessary step to address the links between alcohol and offending described above by aiming to intervene in the often cyclical process of prison admissions where alcohol plays a major part. Effective identification is needed to signpost individuals to appropriate intervention, treatment and support options.

Currently, there are prescribed screening points on admission to all Scottish prisons at which alcohol problems could be identified: reception screening (nurse), medical check (general practitioner) and Core Screen (prison officers). Additionally, prisoners can be referred or self-refer to medical and addiction services at any point during their incarceration. However, questioning on entry for alcohol does not extend much beyond a 'yes/no' response to the question 'Do you have an alcohol problem?' This was recalled by prisoners themselves as an "aye or no" question in the course of qualitative interviews conducted as a separate part of this study [13]. Any further enquiry following a closed question such as this depends on the individual prisoner's response and the professional's interpretation. Furthermore, the question on alcohol is part of a much wider assessment of a range of health and social needs carried out at a stressful and busy time when entering prison. Drinking problems are therefore unlikely to be an immediate concern for individuals at this time, apart from the possible presence of withdrawal symptoms, and so more extensive and validated identification/screening is required for all.

Effective assessment of prisoners is also essential to establish the range of needs relating to alcohol problems, in order to provide adequate, high quality health and social supports to address these needs. Research in England has suggested that only a limited proportion of those with alcohol problems are identified on entry to the prison system [20]. In the Scottish prison system, Graham [14] found disparities between self-reported rates of alcohol problems and recording of clinical diagnosis that "suggest that alcohol problems are under-detected, under-recorded and under-treated in SPS" [[14]:p18]. In England, Newbury-Birch and colleagues [3] also found discrepancy between prevalence of alcohol use disorders (AUDs) detected through screening using AUDIT [21] (score 8+) and prevalence identified by the current OASys (Offender Assessment System) process (part of the National Offender Management System (NOMS)). Research relying on current routine, administrative data sources in UK prisons is therefore likely to underestimate prevalence of alcohol problems.

In a rapid review conducted as part of this study, 13 studies which evaluated the reliability and/or validity of a range of alcohol screening tools with prison populations were identified [13]. The Alcohol Use Disorders Identification Test (AUDIT) was one of three screening tools which emerged as having good reliability with adult prisoners. AUDIT is a 10 question screening tool addressing key areas of alcohol experience as described further below. The AUDIT screening tool is currently being used in the UK for several schemes relating to offenders, for example, to screen offenders for inclusion in Alcohol Arrest Referral Schemes (AARS). It is also the screening tool of choice in a current Scottish pilot study exploring the feasibility and potential effectiveness of alcohol brief interventions (ABI) in the community justice setting (an overview of the Alcohol and Offenders CJS Research Programme is available [22]). In England, AUDIT is recommended as a screening tool for probation officers [23] and in the piloting of a training intervention for Offender Health Trainers (OHTs).

This paper reports on data collected using the AUDIT screening tool with entrants to a Scottish prison. It assesses the extent of alcohol problems in this particular setting and provides additional analysis by key socio-demographic and crime-related factors. The paper also assesses the value and feasibility of using the AUDIT screening tool in prison settings. This work formed part of a larger national study [13] designed to directly inform Scottish policy and practice developments to address the links between alcohol and offenders and to provide high quality healthcare to prisoners in Scotland.

## Methods

A screening questionnaire was developed which incorporated the World Health Organization's AUDIT standardised screening tool [21] and supplementary contextual questions. AUDIT comprises ten questions addressing four areas: alcohol intake; abnormal drinking behaviour and alcohol dependence; the link between alcohol consumption and the detection of psychological effect; and alcohol-related problems. A standard 'drink' (Question 2) was considered to be 8 grammes of pure alcohol equating to 1 unit. An Alcohol Consumption Reckoner was designed which provided a list of culturally sensitive drink types, including pictures and units per glass, can and bottle as appropriate. This aimed to facilitate respondents' calculation of units of alcohol consumed, in order to enhance accuracy and improve the reliability and validity of the information.

Scores from the ten individual AUDIT questions (Additional File 1) are summed to give overall scores ranging from 0-40. Babor et al [[21]:p19] propose that "total scores of 8 or more are recommended as indicators of hazardous and harmful alcohol use, as well as possible alcohol dependence" i.e. the likely presence of an AUD. This is refined to give the following guidance:

• Zone I 0-7 represents low risk drinking or abstinence

• Zone II 8-15 represents a medium level of alcohol problem: ('hazardous' drinking)

• Zone III 16-19 represents a high level of alcohol problem: ('harmful' drinking)

• Zone IV 20-40 clearly warrants further diagnostic evaluation for alcohol dependence: ('possibly dependent')

Eight supplementary questions were added in order to provide additional contextual data for the screening results. These questions enquired into: sentence status, impact of alcohol and substances on the crime, treatment experience, employment, education, marital/family status and age. Showcards facilitated response choices where these were too detailed for the administered questionnaire (see AUDIT and supplementary questions: Additional File 1). The supplementary questions were asked after the AUDIT screen to avoid influencing the screening tool's results.

Screening was undertaken with all new entrants to one male prison in Scotland over a ten week period (n = 259). The prison intake incorporated short term and longer term sentenced prisoners as well as remand, and included young offenders as well as adults. The screening questionnaire was administered at the same time as the Scottish Prison Service (SPS) Core Screen/Induction interview by the four prison officers who routinely undertook this procedure (undertaken in the first few days of entry and typically after the reception screening and general practitioner medical check described above). A preparatory two hour training session was held with these officers, together with relevant management and administrative staff.

The study was conducted according to ethical principles essential in research with vulnerable groups. The research was reviewed by the Scottish Prison Service Research Ethics Committee prior to commencement. In addition, the study was taken through an ethical review at the Institute for Social Marketing, University of Stirling, to ensure additional scrutiny. In response to an initial enquiry, the National Research Ethics Service (NRES) decided an NRES ethics review was not required. All new entrants to the establishment were informed about the aims of the screening, and the study it was part of, and given the choice to participate or not. All respondents were given a leaflet, *'What's in a Drink?*' [24]. Prison service information was added regarding where prisoners could get help with their drinking, if desired. Screening with AUDIT and awareness of what scores indicated problematic drinking augmented the officers' routine practice.

Data collation and input was the responsibility of the research team. Anonymised data were sent confidentially and securely to researchers every week by the prison-based administrator and checked for errors and consistency. Overall 259 screening questionnaires collected between November 2009 and January 2010 were eligible for inclusion in the final analysis. This represents 88% of overall admissions to the study prison during the screening period (recorded at 294): there were four refusals recorded and some admissions would not have gone through the Core Screening due to the circumstances of their admission.

Data were analysed using PASW (Predictive Analytics Software) Statistics - formerly Statistical Package for the Social Sciences (SPSS) - version18. Descriptive statistics were produced and analysis of variance (ANOVA) was used to test for differences in the mean scores of two or more groups. To facilitate further analysis of AUDIT Question 1 (How often do you have a drink containing alcohol?) frequency of drinking was recoded to indicate approximate frequency per week (0 = never; 0.25 = monthly or less; 0.5 = 2-4 times a month; 2.5 = 2-3 times per week; 4 = 4 or more times a week).

## Results

### Demographic and custody-related information

Demographic measures are summarised in Table 1. This was a relatively youthful sample of adult male prisoners: the majority of respondents were under 30 years of age (62%) including 36% who were less than 25 years old. Mean and median ages were 29 and 27 years respectively. Further information showed strong indications of socio-economic deprivation and social exclusion among the sample, providing a picture of men living outside a range of social support mechanisms. The majority of respondents (75%) were unemployed, although 14% described themselves to be in full-time employment. In addition, over two-fifths (41%) reported having no educational qualifications, with a further two-fifths (42%) having only basic qualifications of Standard Grades or NVQs at Foundation or Intermediate levels or equivalents. Examining family status, nearly two-thirds (61%) described themselves as single, while around one third reported being in a co-habiting relationship (29%), and only 3% described themselves as married. Almost two-thirds (60%) of those who answered reported having children, a markedly higher proportion than those reporting a co-habiting relationship.

**Table 1:** Age and socio-economic indicators

*Base: All respondents answering relevant question*		
*^1^Base: 259*		
*^2^Base: 257*		
*^3^Base: 258*		
*^4^Base: 258*		
*^5^Base: 247*	%	*(no)*
**Age of respondents**^1^		
18-24 years	36	*(94)*
25-29 years	26	*(67)*
30-39 years	25	*(64)*
40-64 years	13	*(34)*

**Employment status before prison**^2^		
Unemployed/benefits	75	*(193)*
Full-time employment	14	*(35)*
Part-time employment	3	*(7)*
Casual employment	4	*(9)*
Full-time education/training	2	*(5)*
Other	3	*(8)*

**Educational qualifications**^3^		
None of these qualifications	41	*(106)*
Standard Grade or equivalent	22	*(58)*
GNVQ/GSVQ Foundation or Intermediate or equivalent	20	*(51)*
Higher Grade or equivalent	4	*(11)*
GNVQ/GSVQ Advanced or equivalent	6	*(16)*
HNC, HND, SVQ Level 4, RSA Advanced Diploma or equivalent	3	*(9)*
First Degree, Higher Degree, SVQ Level 5 or equivalent/professional qualifications	3	*(7)*

**Relationships**^4^		
Single	61	*(158)*
Living with partner	29	*(75)*
Married	3	*(7)*
Divorced	3	*(7)*
Other	4	*(11)*

**Number of children**^5^		
No children	40	*(99)*
1 child	28	*(70)*
2 children	16	*(40)*
3 children	10	*(24)*
4+ children	6	*(14)*

Self-reporting of offences showed that 31% of reasons for detention related to dishonesty (including theft, shoplifting and housebreaking); 27% to violent crime (predominantly 'serious assault & attempted murder'); and 24% to other crimes (including 'crimes against public justice', drugs, and 'handling an offensive weapon') (Table 2: categories are based on the classification of crimes and offences used in Prison Statistics Scotland [25]).

**Table 2:** Respondent 'current offence' categories (only/main category^1^)

**Categories**^**2**^	Total *(n=259)*		Sentenced *(n=122)*		Remand *(n=137)*	
	%	*(no)*	%	*(no)*	%	*(no)*
Dishonesty (inc. theft, shoplifting, housebreaking)	31	*(79)*	35	*(43)*	26	*(36)*
Violence (predominantly 'serious assault & attempted murder')	27	*(70)*	22	*(27)*	31	*(43)*
Other crimes (inc. 'crimes against public justice', drugs, 'handling an offensive weapon')	24	*(62)*	21	*(26)*	26	*(36)*
Miscellaneous offences	9	*(23)*	11	*(13)*	7	*(10)*
Motor vehicle offences	3	*(9)*	[-]	(*)	[-]	(*)
Indecency	[-]	(*)	[-]	(*)	[-]	(*)
Fireraising	[-]	(*)	[-]	(*)	0	*(0)*
No information/no category	5	*(12)*	[-]	(*)	[-]	(*)

* Indicates values that have been suppressed due to the potential risk of disclosure and to help maintain prisoner confidentiality

^1 ^Takes the 'highest' category where more than one given; 55 (21%) reported more than 1 category, including 4 who reported more than 2 categories.

^2 ^Categories based on the classification of crimes and offences used in Prison Statistics Scotland (Scottish Government 2009b)

Other aspects of current and previous prison experience are shown in Table 3. The majority of offenders were on remand or had short term sentences, categories which have limited access to alcohol interventions in Scottish prisons [13,14] (53% on remand and 29% and 51% of those sentenced being sentenced to less than 6 months and 6-24 months respectively: the latter representing 12% and 24% of the total sample). In addition, a considerable majority (88%) had been in prison before, further emphasising service provision challenges and opportunities.

**Table 3:** Sentence status and prison experience

*Base: All respondents answering relevant question*		
*^1 ^Base: 259*		
*^2 ^Base: 117*		
*^3 ^Base: 259*	%	*(no)*
**Sentence status**^1^		
Sentenced	47	*(122)*
Remand	53	*(137)*

**Length of sentence (among sentenced)**^2^		
31 days or under	[-]	*(^α^)*
Less than 3 months	5	*(6)*
3 months - less than 6 months	24	*(29)*
6 months - less than 2 years	51	*(62)*
2 years - less than 4 years	11	*(13)*
4 years or over/Life	[-]	*(^α^)*

**Previous prison experience^3^**		
Yes	88	*(228)*
No	12	*(31)*

*^α ^*Indicates values that have been suppressed due to the potential risk of disclosure and to help maintain prisoner confidentiality

### Prevalence of alcohol-related problems as indicated by AUDIT scores

The overall AUDIT scores across all respondents show a high prevalence of alcohol problems among these adult male prisoners (Table 4). Nearly three-quarters of respondents had scores indicating an AUD (73%) as indicated by an AUDIT score of 8+ [21], including over a third of respondents (36%) having scores in Zone IV indicating possible dependence (20-40, see methods section).

**Table 4:** AUDIT scores of alcohol-related problems: total scores and by behavioural measures

*Base: All respondents (259)*					%	*(no)*
0-7 Zone I					27	*(70)*
8-15 Zone II					27	*(71)*
16-19 Zone III					9	*(24)*
20-40 Zone IV					36	*(94)*

**Behavioural Measures^1^**	**Score (%)**					***Base***^***1***^
		
	**0**	**1**	**2**	**3**	**4**	

Q1 - How often drink	15	29	14	21	21	*259*
Q2 - How many drinks typical drinking day	2	5	5	6	83	*221*
Q3 - How often 6+ units	10	22	18	30	21	*221*
Q4 - How often can't stop	49	11	10	13	17	*221*
Q5 - How often failed expectations	54	16	12	8	10	*221*
Q6 - How often need drink in morning	69	9	4	5	12	*221*
Q7 - How often feel guilt or remorse	48	16	15	12	8	*221*
Q8 - How often can't remember	32	27	16	15	9	*221*
Q9 - How often injured self or other person	26	-	31	-	43	*259*
Q10 - How often suggested you cut down	54	-	12	-	33	*259*

^1 ^Full questions and available response scores given in Additional File 1

^2 ^For Qs2-8, base = 221: 38 prisoners were not asked these questions as they reported that they never drink alcohol

Additional analysis showed that 25 respondents (11% of drinkers) reported positively that they were 'currently in treatment in relation to their drinking'. This represents a quarter of those whose AUDIT scores suggest further assessment of treatment needs on the basis of their experiences before prison entry (i.e. 27% of the 94 respondents with AUDIT scores of 20-40, 'possibly dependent'). Responses from those 'in treatment' suggest that for around one third (n = 8), the 'treatment' they reported was instigated during this current detention with the remaining 17 respondents (18% of those with AUDIT scores of 20-40) reporting having attended a range of local community-based alcohol-related agencies. These figures need to be viewed with caution as respondents' interpretation of the question may be variable, and there is potential for subsequent referrals to services further into their admission after the screening process. Nevertheless, since the AUDIT questions relate to behaviour prior to prison entry the level of prior engagement with services is arguably low.

### Examination of behavioural AUDIT measures

The AUDIT scores were further examined by the individual behavioural measures as also shown in Table 4.

#### Alcohol intake: Questions 1-3

For a considerable proportion of the total sample, drinking was a regular part of their lives, with 21% saying they drank four or more times a week and a further 21% drinking two to three times a week (Q1). However, 15% of respondents said they had never drunk in the past year. Response to heaviness of drinking (Q2) shows that drinking a high number of units of alcohol in a session is common among the subsample who drank in the last year (n = 221), with 83% saying they would drink 10 or more drinks (units) on a 'typical' drinking day (the UK government weekly drinking guidelines are that men should not regularly drink more than 3-4 units a day [26]). Examining how often the sample (excluding non-drinkers) tended to drink 6 or more units (Q3), over half (51%) reported drinking at these levels at least weekly, including 21% reporting drinking that amount daily or almost daily.

#### Indications of presence or incipience of alcohol dependence: Questions 4-6

Overall, around half the sample who drank identified with two of the questions (Q4 and Q5). For example, 51% said they felt they could not stop drinking once started (with around 30% saying this was weekly-daily), and 46% said that they had failed to do what was normally expected from them because of drinking (with 18% saying this was weekly-daily). Almost one third reported needing a first drink in the morning to get themselves going after a heavy drinking session (Q6: 31%, with 17% saying this happened weekly-daily).

#### Harm from drinking: Questions 7-10

Reported feelings of guilt or remorse after drinking during the last year were relatively low among drinkers, with 48% overall saying they had never felt such feelings, in spite of the high drinking levels reported. Interestingly, even fewer among heavy drinkers reported guilt or remorse (20% of drinkers with a Zone IV score). Around two-thirds (67%) reported being unable to remember what happened the night before because they had been drinking, although again this tended to be intermittent, with 27% responding less than monthly, although 9% saying it was on a daily or almost daily level.

Two-fifths of the total sample (43%) said they or someone else had been injured as a result of their drinking during the last year, although the question does not define the nature of the injury nor any link with violence (Q9). A further 31% said that injuries related to their drinking had been experienced in previous years. Finally, nearly half of respondents (46%) said that a relative or friend or a doctor or another health professional had been concerned about their drinking or suggested they cut down (Q10), including 33% saying this had happened during the last year.

### Comparison by age group

Analysis of Variance (ANOVA) found significant differences, by age, in the mean AUDIT scores (see Table 5). The oldest age group (40-64 years) had a higher mean AUDIT score of 20.9 (sd = 13.7) compared with 30-39 year-olds, who had a mean AUDIT score of 12.2 (sd = 11.6) (p < 0.05). In addition, the proportion of those with Zone IV scores of 20-40 was high among 18-24 year-olds (40%) as well as among 40-64 year-olds (56%).

**Table 5:** AUDIT score by age category

*Base: All respondents*	18-24 **years** *(n=94)*		25-29 years *(n=67)*		30-39 years *(n=64)*		40-64 years *(n=34)*		Total *(n=259)*	
	%	*(no)*	%	*(no)*	%	*(no)*	%	*(no)*	%	*(no)*
0-7 Zone I	17	*(16)*	27	*(18)*	45	*(29)*	21	*(7)*	27	*(70)*
8-15 Zone II	32	*(30)*	[-]	*(^α^)*	[-]	*(^α^)*	24	*(8)*	27	*(71)*
16-19 Zone III	11	*(10)*	[-]	*(^α^)*	[-]	*(^α^)*	0	*(0)*	9	*(24)*
20-40 Zone IV	40	*(38)*	31	*(21)*	25	*(16)*	56	*(19)*	36	*(94)*

Mean *(SD)*	16.6	*(9.8)*	16.0	*(11.5)*	12.2*	*(11.6)*	20.9*	*(13.7)*	15.9	*(11.5)*

*^α ^*Indicates values that have been suppressed due to the potential risk of disclosure and to help maintain prisoner confidentiality

* The mean difference is significant at *P *< .05

Analysis of responses to individual questions by age groups also reflected these differences. Notably in relation to Question 1 (reported frequency of having a drink containing alcohol),18-24 year-olds most commonly reported drinking 2-3 times a week (32%), whilst 40-64 year-olds most commonly reported drinking 4 or more times a week (41%), thus drinking more frequently than the other three main age groups (see Table 6). In contrast, frequency levels among 30-39 year-olds were markedly lower, with over a quarter never drinking in the past year (28%; accounting for nearly half those never drinking). To facilitate comparison by age, frequency of drinking was recoded to give approximate frequency per week (0 = never; 0.25 = monthly or less; 0.5 = 2-4 times a month; 2.5 = 2-3 times per week; 4 = 4 or more times a week). ANOVA indicated that frequency of drinking was higher among 40-64 year-olds (mean = 2.4, sd = 1.6) compared with 30-39 year-olds (mean = 1.2, sd = 1.6, p < 0.05) and compared with 25-29 year-olds (mean = 1.3, sd = 1.6, p < 0.05).

**Table 6:** Reported frequency of having a drink containing alcohol across age groups: response to AUDIT Question 1

*Base: All respondents*	18-24 years *(n=94)*		25-29 years *(n=67)*		30-39 years *(n=64)*		40-64 years *(n=34)*		Total *(n=259)*	
	%	*(no)*	%	*(no)*	%	*(no)*	%	*(no)*	%	*(no)*
**Drink frequency **(allocated scores^1^)										
Never (0)	10	*(9)*	[-]	*(^α^)*	28	*(18)*	[-]	*(^α^)*	15	*(38)*
Monthly or less (0.25)	29	*(27)*	[-]	*(^α^)*	28	*(18)*	[-]	*(^α^)*	29	*(75)*
2-4 times a month (0.5)	14	*(13)*	15	*(10)*	13	*(8)*	18	*(6)*	14	*(37)*
2-3 times a week (2.5)	32	*(30)*	13	*(9)*	13	*(8)*	24	*(8)*	21	*(55)*
4 or more times a week (4)	16	*(15)*	19	*(13)*	19	*(12)*	41	*(14)*	21	*(54)*

Mean *(SD)*	1.6	*(1.5)*	1.3*	*(1.6)*	1.2*	*(1.6)*	2.4*	*(1.6)*	1.5	*(1.6)*

^1 ^Means derived by allocating scores from 0, never, to 4, 4 or more times a week

*^α ^*Indicates values that have been suppressed due to the potential risk of disclosure and to help maintain prisoner confidentiality

* The mean difference is significant at *P *< .05

For those with the highest AUDIT score range (Zone IV 20-40), notable differences in behaviours are also apparent by age. For example, the mean score for the youngest respondents (18-24 year-olds) is 27, with mean scores gradually increasing with age to 32 for those in the 40-64 years age band. Furthermore, whilst the AUDIT guide suggests that a Zone IV score of 20-40 indicates likelihood of dependent drinking, examination of age breakdown reveals differing patterns of drinking in this sub-sample similar to the overall sample. Taking as a start point that nearly all with Zone IV scores (98%) drink heavily on a typical drinking day (10 or more drinks Q2), older drinkers with Zone IV scores, especially 40-64 year-olds, tended to be more frequent and more dependent drinkers, compared with their 18-24 year old counterparts. For example, as a key indicator of dependence (Q6), over half of 40-64 year-olds (10 of 19 respondents: 53%) experienced needing a drink in the morning after a heavy drinking session on a daily or almost daily basis whilst the youngest respondents (18-24 year-olds) tended not to report this with 22 of 38 respondents (58%) saying this never happened.

In addition, in the youngest age band (18-24 year-olds) the greatest proportion (55%) reported drinking 2-3 times a week (21 of 38 respondents), whereas older respondents were more likely to drink 4 or more times a week, increasing with age to 14 of the 19 (74%) 40-64 year-olds reporting drinking in this way (Q1). Finally, younger respondents were more likely to drink 6+ units on a weekly basis (23 of 38 respondents (61%)) compared with respondents 25 years and older, who were more likely to be daily or almost daily drinkers at this level (Q3).

### Association between drinking and crime

Respondents' beliefs about whether alcohol was a factor in their index offence, i.e. the reason for their current prison admission, were explored during supplementary questioning. Table 7 shows that two-fifths (40%) of respondents reported that alcohol was a factor with a further 5% acknowledging they had been drinking at the time of their offence. This was most notable among the 40-64 year-olds followed by 18-24 year olds (56% and 44% respectively). However, there was no significant difference by age (p > 0.05). Further analysis among those who said that they had not drunk at all in the previous year (Q1, n = 38: 15% of total sample) showed that nearly all (97%: n = 37) had been in prison before, perhaps suggesting abstinence in response to past problematic drinking and possibly linked with crime. In addition, among those reporting being sentenced for violent crimes (n = 70), the proportion linking their drinking and the offence was significantly higher than for those reporting other types of crime (50% vs. 36%, p < 0.05).

**Table 7:** Alcohol reported as a factor in offence by age

*Base: All respondents*	18-24 years *(n=94)*		25-29 years *(n=67)*		30-39 years *(n=64)*		40-64 years *(n=34)*		Total *(n=259)*	
	%	*(no)*	%	*(no)*	%	*(no)*	*%*	*(no)*	%	*(no)*
Yes	44	*(41)*	37	*(25)*	28	*(18)*	56	*(19)*	40	*(103)*
No, was sober	51	*(48)*	[-]	*(^α^)*	[-]	*(^α^)*	[-]	*(^α^)*	55	*(143)*
No, but had been drinking	5	*(5)*	[-]	*(^α^)*	[-]	*(^α^)*	[-]	*(^α^)*	5	*(13)*

*^α ^*Indicates values that have been suppressed due to the potential risk of disclosure and to help maintain prisoner confidentiality

Among those who reported that alcohol was a factor in the index offence, nearly half (49%) of those who responded to a supplementary question (n = 90) agreed that drugs were also involved in the offence. An additional eight respondents (9%) who reported drinking at the time, but did not think alcohol was a factor in the offence, volunteered that they had also taken drugs. This indicates a relatively prevalent influence of mixed substance use.

Comparison of AUDIT scores indicates further links between alcohol and the index offence. For example, the proportion of those with Zone IV AUDIT scores reporting alcohol to be a factor in the offence was significantly higher than those with Zone I-III scores (76%: n = 71 vs. 19%: n = 32, p < 0.001). Similarly, the proportion of those with Zone IV AUDIT scores reporting violent crimes was significantly higher than those with Zone I-III scores (39%: n = 36 vs. 22%: n = 34, p < 0.01).

Assessing AUDIT scores by sentence status shows that a slightly higher proportion of sentenced prisoners had Zone IV scores than remand prisoners (39% vs. 34%) and a smaller proportion had Zone I scores (21% vs. 32%). Analysis by sentence length (Table 8) showed that AUDIT scores tended to be higher among those whose sentences were shorter (e.g. 45% with sentences less than six months had Zone IV scores). However, there was no significant difference by sentence length (p > 0.05).

**Table 8:** AUDIT score by sentence length

*Base: All sentenced (117)*	< 6 months *(n = 38)*		6 months to < 2 years *(n = 62)*		2+ years & Life *(n = 17)*
	%	*(no)*	%	*(no)*	*(no)*
0-7 Zone I	13	*(5)*	23	*(14)*	*(5)*
8-15 Zone II	29	*(11)*	31	*(19)*	*(7)*
16-19 Zone III	13	*(5)*	[-]	*(^α^)*	*(^α^)*
20-40 Zone IV	45	*(17)*	[-]	*(^α^)*	*(^α^)*

Mean *(SD)*	19.9	*(12.1)*	16.2	*(11.1)*	*12.9 (11.1)*

*^α ^*Indicates values that have been suppressed due to the potential risk of disclosure and to help maintain prisoner confidentiality

## Discussion

### Behaviour patterns and demographic contexts

To the best of our knowledge, this is the first alcohol screening study to explore differing drinking patterns among prisoner sub-groups using AUDIT, thus identifying a need for differing intervention approaches. Highest levels of consumption and AUDIT scores indicating 'possible dependence' are most apparent among both 18-24 and 40-64 year-olds. However, younger drinkers were less likely to demonstrate habitual and addictive behaviours compared to the older age group; for example they were less likely to drink daily or to need a first drink in the morning to get going after a heavy drinking session. Thus younger heavy drinkers are likely to have differing support needs and are arguably more unlikely to identify themselves as having a 'problem' in response to a limited 'aye or no' screening question. In addition, whilst in the general population, the proportion indicating possible problem drinking by agreeing with two or more items in CAGE consistently falls with age [15], in our prisoner sample the trend for decline in AUDIT scores with age is interrupted by an increase in scores indicating AUDs among 40-64 year-olds (however the former survey has a broader age range than our study and uses CAGE rather than AUDIT). The SPS Scottish Prisoner survey also identified high levels of problematic use across age groups, rather than a gradual decline with age [13]: for example, among male prisoners, 53% of 16-24 year-olds answered two or more CAGE questions positively and 47% of 45-54 year-olds indicated having an alcohol problem in this way.

Furthermore, universal screening using AUDIT has highlighted a marked prevalence of high consumption levels and harmful/hazardous/dependent drinking behaviours among male prisoners prior to entry to the study prison (73% having scores indicating AUDs, including 36% 'possibly dependent'). This confirms the potential for prisons as a setting for tackling alcohol misuse and the importance of rapid access to appropriate interventions. Similar high levels of alcohol problems are identified in other recent studies in the criminal justice setting using AUDIT (e.g. LG unpublished data and Newbury-Birch and colleagues [3]). Results also show consistency with self-reporting identified in the SPS Scottish Prisoner Survey using the CAGE screening tool [13]; for example 44% male prisoners gave responses indicating likely problematic use, compared with 36% Zone IV AUDIT scores indicating 'possible dependence'.

Prisoner alcohol consumption levels appear considerably higher than in the male general population. Although not directly comparable, 2009 Scottish Health Survey (SHeS) figures suggest that 26% of men (16 years and over) in the general population drink over eight units on their 'heaviest' drinking day of the week [[15]:p94], whereas 83% of prisoner respondents who drank in this sample said they consumed 10 or more units on a 'typical drinking day'. As a further comparison between the general population and prisoner drinking experience, the SHeS suggests 14% of males agreed with two or more items in a modified CAGE screening tool, indicating possible 'problem drinking' [[15]:p97], markedly lower than the 44% of male respondents indicating problematic drinking in the SPS Scottish Prisoner Survey which also used a CAGE screening tool as above [13].

The proportion of non-drinkers in the offender sample (15% over the previous year) is higher than in the general population, particularly in 'middle' age group (28% of 30-39 year-old prisoners). For example, the 2009 SHeS, showed 10% of males reporting not drinking, with the highest proportions of non-drinkers among 65 and over age groups, not represented in our prison sample [15,27]. However, the SHeS results also show that non-drinking among males is most prevalent in the lowest income quintile (20%), a demographic most comparable with offender populations, suggesting our sample may not be atypical among peers living in disadvantaged communities [15,28]. It is also possible that some non-drinkers were abstaining in response to previous alcohol problems, perhaps reflected in the vast majority having previous prison experience, and they may still need support with alcohol-related issues.

The findings also highlight the high proportion of prisoners on remand or on very short sentences which presents further challenges to service provision, requiring a rapid response when in prison and greater attention to care pathways facilitating access to community-based interventions.

### Association with crime

Drinking alcohol was self-reported as associated with the index crime among two-fifths of respondents. This was most notable among older and younger prisoners, and was also higher among the sub-sample reporting violent offences (50%, significantly higher than those reporting other types of crime). This is similar to responses to the SPS 2009 survey [29] where half of respondents reported being drunk at the time of their offence, an increase of 10% on 2005 figures of 40%. McKinlay and colleagues have similarly highlighted the growing influence of alcohol on offending among young offenders [4]. For example the proportion that blamed their current offence on drinking rose from 30% in 1979 to 40% in 1996 and 57% in 2007. In addition, the proportion of those with high AUDIT scores (Zone IV) who reported alcohol to be a factor in the crime and who reported violent offences was significantly higher than those with Zone I-III scores.

Whilst it would be simplistic to identify alcohol as the only factor in these crimes, the findings add to the argument for addressing alcohol issues as a priority in the criminal justice setting, and their potential impact on reducing recidivism. The combined influence of drugs is also likely to be a factor but it is important that alcohol is addressed independently as needed.

### Disparity with access to treatment and support

The data provide indications of disparity between the high levels of harmful/hazardous/dependent drinking identified and low levels of engagement with 'treatment' in this study population. Only around a quarter of those with AUDIT scores indicating possible dependency reported being 'in treatment for their drinking', incorporating even fewer having been engaged in ongoing community based work with alcohol issues. Whilst the data need to be viewed with caution as respondents' interpretation of the question appears variable and there is also scope for referral to services during the prison admission, nevertheless the proportion reporting existing engagement with services is low, considering the AUDIT scores relate to behaviour prior to admission.

The challenging gap between prevalence of high consumption and problematic behaviours, and the current levels of service provision and access to alcohol interventions within prisons is reflected across the prison estate. The annual SPS survey data [13], show that in the context of high prevalence of reported alcohol problems only around one third (31%) of prisoners said they had been assessed for alcohol use on admission to prison, and an equal proportion (31%) said they had been given a chance to receive treatment during their sentence, although only one fifth (19%) said they *had *received help/treatment. More positively, over one third of prisoners said they would take help for alcohol problems in prison (39%) and outside prison (36%), if offered.

### Value and feasibility of use of AUDIT as a screening tool

These findings confirm the potential of the AUDIT screening tool in terms of its value and feasibility in criminal justice settings. However, this analysis has also revealed important variations based on individual questions, particularly in revealing variations in drinking behaviour patterns and dependency levels among those with high levels of consumption, and also the presence of non-drinkers. Thus, in identifying individual and service needs, attention to individual question responses is required which in turn could enhance the value of using AUDIT.

Using a validated screening tool on entry to prison is of key importance in identifying individual needs and appropriate routes linked to care pathways, as well as a clearer understanding of service requirements. Limited 'aye or no' questioning on admission such as 'Do you have an alcohol problem?' is likely to meet with the answer 'no', as shown from qualitative enquiry with prisoners and staff as a separate part of this study [13]. A 'no' response was felt to be likely for a range of reasons, for example questioning on entry is at a time when prisoners are faced with questions on a whole range of issues and additional competing concerns are likely to take precedence over drinking issues, making alcohol problems less of an immediate concern apart from any withdrawal needs. Indeed prisoners might still be 'under the influence' of drink and/or drugs at this point. In addition, individuals may be reluctant to acknowledge alcohol problems or want to deal with them. In our study, using AUDIT allowed greater depth of exploration in a structured and non-threatening way, but also prison officers' experience suggested that opportunities for further discussion were created.

Finally, the administration of the AUDIT screening tool by trained prison officers as part of routine procedures was successful, including collection of the additional demographic data.

### Links with disadvantage and exclusion

The screening highlighted indicators of disadvantage and social exclusion among prisoners, with a high proportion of men without employment, with limited educational achievements and living alone. These findings contribute to a picture of men tending to live outside a range of social support mechanisms such as living with partners and parenting. Lack of social support has major implications for successful resettlement and desistence from offending [30], although it may be difficult to know whether less problematic drinkers are more likely to attract and retain a partner, or whether they drink less because they have a partner or children. In addition high levels of literacy problems, indicated by low education attainment levels, can have an impact on access to services and health information as well as employment and other inclusion opportunities [13,31,32]. These issues are especially acute among those with limited stays in prison, either on remand or very short sentences.

### Study limitations

There are some limitations to applying the study findings more widely; for example women prisoners (a small minority in Scotland) were not included as this was a single prison study. In addition, compared to the general Scottish prison population over a similar period, the sample is somewhat younger with shorter sentences [25]; for example 36% of respondents were under 25 years old compared with 28% of males in custody in Scotland, and 32% of respondents had sentences of less than 6 months compared with 8% across the prison population. This reflects the varied functions of different establishments across the Scottish prison estate. Nevertheless, youthful drinkers and related problematic drinking behaviours are also of concern in the wider population, as well as older dependent drinkers. In addition, the study benefits from incorporating repeat and shorter stay 'revolving door' offenders, as well as longer term and older prisoners. The high proportion of those with prior prison experience (88%) resonates with the 'Scotland's Choice' report which highlights that in 2006/07 nearly one in six of the 7,000 offenders who received a custodial sentence had already been to prison on more than ten previous occasions [[31]:p57].

The screening timing on entry may have resulted in underestimates of prevalence. Maggia et al [33] identified consistently raised AUDIT scores when comparing individual offenders' responses on entry and around 15 days later. Although the screening exercise occurred soon after the routine general health checks undertaken by a nurse and a general practitioner respectively, it seems unlikely this would have had an effect on responses, given that prisoners themselves indicated the questioning during these contacts was very limited [13]. Furthermore, the data collection approach means there is no collateral assessment of the nature of individuals' drinking behaviour or comparison with other scores. However, the AUDIT scores obtained are consistent with other offender studies [3,29]. There is also reliance on offender self-report in relation to crimes and sentencing and other socio-demographic details, without cross-checking with other records. However, a previous study showed 80% concordance between self-reported convictions and official records [34]. In addition, the sample size is relatively small (n = 259), limiting statistical analysis, particularly for sub-populations within the sample.

## Conclusions

As far as the authors are aware, this is the first study in offender populations to detect differences in drinking patterns between younger and older 'possibly dependent' drinkers (score 20-40), reflecting AUDIT scores and analysis of individual question responses. The screening tool highlights varying needs among those with high scores and also enables identification of those who might not acknowledge that they have an alcohol problem in response to a limited 'aye or no' screening question, for example younger binge drinkers with few indications of dependency. This in turn creates greater opportunities to encourage engagement with interventions. In addition, the findings confirm the value and feasibility of routine use of the AUDIT screening tool in prison settings to considerably enhance practice in the detection and understanding of alcohol problems, improving on current more limited questioning ('aye or no' questions).

The high prevalence of problematic drinking identified in the study, and the varied patterns of heavy drinking behaviours, together with links between drinking and crime and recidivism, support the argument for more extensive provision of alcohol-focused interventions in prison and related criminal justice settings. There is a need for a tiered approach, varied in intensity, and carefully targeted based on effective initial screening and assessment. The need for a rapid response and pathways providing links with community-based services is highlighted by the high proportion of those on remand or sentenced for very short periods and the high proportion of repeat offenders. Throughcare, outreach and inreach are essential concurrent developments that would help develop more streamlined and consistent care pathways. Potential interactions between drinking and drug use also need to be taken into account, in addition to other complex needs such as mental health, but the need for more alcohol specific interventions should also be prioritised. Finally, the high prevalence of socio-demographic indicators of disadvantage has implications for both successful desistance and rehabilitation, and holistic interventions which address such broader social and contextual issues are urgently required, which in turn may address prevalence of alcohol problems.

## Abbreviations

AARS: Alcohol Arrest Referral Schemes; ABI: alcohol brief intervention; ANOVA: analysis of variance; AUDIT: Alcohol Use Disorders Identification Test; AUD: alcohol use disorder; CAGE: 'cut down, annoyed, guilty, eye-opener'; CJS: Criminal Justice System; NHS: National Health Service; NOMS: National Offender Management System; NRES: National Research Ethics Service; NVQ: National Vocational Qualification; OASys: Offender Assessment System; OHT: Offender Health Trainers; p/P: probability; PASW: Predictive Analytics Software; Q: question; sd/SD: standard deviation; SHeS: Scottish Health Survey; SPS: Scottish Prison Service; SPSS: Statistical Package for the Social Sciences; UK: United Kingdom; WHO: World Health Organization.

## Competing interests

The authors declare that they have no competing interests.

## Authors' contributions

SM and TP developed the detailed design of this study, and the final research instruments. LG and AM devised the overall research programme, including the original design for this study, and actively commented on design details, the report and this paper. With TP leading the overall study, SM led the section reported here, including data collection, overseeing training of officers and reporting of the analysis. OB oversaw data input and contributed to the analysis and reporting. AB undertook the statistical analyses and contributed to reporting. All authors read and approved the final manuscript.

## Pre-publication history

The pre-publication history for this paper can be accessed here:

http://www.biomedcentral.com/1471-2458/11/865/prepub

## Supplementary Material

Additional file 1 **AUDIT screening questionnaire and supplementary questions**. This file contains the screening tool used in the study; comprising the AUDIT screening questionnaire^1 ^and eight supplementary questions designed to provide additional contextual data for the screening results. **^1^**Babor TF, Higgins-Biddle JC, Saunders JB, Monteiro MG: *AUDIT: The Alcohol Use Disorders Identification Test - Guidelines for Use in Primary Care (2^nd ^edition)*. Geneva: World Health Organization; 2001. Click here for file
