# Supplementary material for: Assessment of alcohol problems using AUDIT in a prison setting: more than an 'aye or no' question
Source: BMC Public Health. 2011 Nov 14;11:865. doi: 10.1186/1471-2458-11-865 (PMC3295750; doi:10.1186/1471-2458-11-865)
Supplement: Additional file 1 — AUDIT screening questionnaire and supplementary questions. This file contains the screening tool used in the study; comprising the AUDIT screening questionnaire1 and eight supplementary questions designed to provide additional contextual data for the screening results. 1Babor TF, Higgins-Biddle JC, Saunders JB, Monteiro MG: AUDIT: The Alcohol Use Disorders Identification Test - Guidelines for Use in Primary Care (2nd edition). Geneva: World Health Organization; 2001. [file 1471-2458-11-865-S1.DOC]

**Additional File 1 - AUDIT screening questionnaire^1^ and supplementary questions**

Are you willing to take part in a brief survey about your use of alcohol?

This will be completely anonymous and participation is voluntary.

Yes 🗆 No 🗆 *(Please record response and signature in separate list)*

| **AUDIT: Interview Version**  Read questions as written. Record answers carefully. Begin the AUDIT by saying  *“Now I am going to ask you some questions about your use of alcoholic drinks during this past year.”*  For example, cider, vodka, beers, wines, lagers etc (show list)  Code answers in terms of “standard drinks”. Place the relevant answer number in the box at the right.  **NB. Questions relate to most recent time outside in the community**  ***Thinking about your most recent time in the community.....*** | |
| --- | --- |
| 1. How often do you have a drink containing alcohol?  (0) Never [Skip to Qs 9-10]  (1) Monthly or less  (2) 2 to 4 times a month  (3) 2 to 3 times a week  (4) 4 or more times a week | 6. How often during the last year have you needed a first drink in the morning to get yourself going after a heavy drinking session?  (0) Never  (1) Less than monthly  (2) Monthly  (3) Weekly  (4) Daily or almost daily |
| 2. How many drinks containing alcohol do you have on a typical day when you are drinking?  (0) 1 or 2 *Please write in drinks*  (1) 3 or 4  (2) 5 or 6  (3) 7, 8, or 9  (4) 10 or more | 7. How often during the last year have you had a feeling of guilt or remorse after drinking?  (0) Never  (1) Less than monthly  (2) Monthly  (3) Weekly  (4) Daily or almost daily |
| 3. How often do you have six or more drinks on one occasion?  (0) Never  (1) Less than monthly  (2) Monthly  (3) Weekly  (4) Daily or almost daily  *Skip to Questions 9 and 10 if Total Score*  *for Questions 2 and 3 = 0* | 8. How often during the last year have you been unable to remember what happened the night before because you had been drinking?  (0) Never  (1) Less than monthly  (2) Monthly  (3) Weekly  (4) Daily or almost daily |
| 4. How often during the last year have you found that you were not able to stop drinking once you had started?  (0) Never  (1) Less than monthly  (2) Monthly  (3) Weekly  (4) Daily or almost daily | 9. Have you or someone else been injured as a result of your drinking?  (0) No  (2) Yes, but not in the last year  (4) Yes, during the last year |
| 5. How often during the last year have you failed to do what was normally expected from you because of drinking?  (0) Never  (1) Less than monthly  (2) Monthly  (3) Weekly  (4) Daily or almost daily | 10. Has a relative or friend or a doctor or another health worker been concerned about your drinking or suggested you cut down?  (0) No  (2) Yes, but not in the last year  (4) Yes, during the last year |
| Record total of specific items here | |

**^1^**Babor TF, Higgins-Biddle JC, Saunders JB, Monteiro MG: *AUDIT: The Alcohol Use Disorders Identification Test – Guidelines for Use in Primary Care (2^nd^ edition)*. Geneva: World Health Organization; 2001.

**And now I would like to ask you some brief questions about yourself – again this will be completely anonymous.**

1. **What is your sentence status:**

*– which best describes you?*

Sentenced 🗆 Remand (unconvicted) 🗆 Remand (convicted awaiting sentence) 🗆

What is your current offence (charge / conviction)? ………………………..................................................................................……

IF SENTENCED, **How long is your sentence?** ………………………..................................................................................……

**2) Have you ever been in prison before?**

Yes 🗆 No 🗆 Don’t know 🗆

**3) Do you believe alcohol was a factor in the offence for which you are here?**

Yes 🗆 No, was sober 🗆 No, but had been drinking 🗆 Don’t know 🗆

IF YES, was misuse of illegal or prescription drugs also involved? Yes 🗆 No 🗆

**4) Are you currently in treatment in relation to your drinking?**

Yes 🗆 No 🗆 Don’t know 🗆

IF YES, **can you provide details?** ………………………..................................................................................……

**5) What was your working situation before coming into prison?**

***Showcard 1*** *– which one best describes you?*

Full time employment 🗆 Part-time employment 🗆 Casual 🗆

Training schemes 🗆 Unemployed / on benefits, e.g. incapacity etc 🗆

Full time education 🗆 Other 🗆

**6) Please tell me which, if any, educational qualifications you have, looking at this card.**

***Showcard 2*** *– point to any that apply to you.*

1🗆 2🗆 3🗆 4🗆 5🗆 6🗆 7🗆 8🗆 9🗆 10🗆 11🗆 12🗆

**7) How would you describe your marital/family status?**

***Showcard 3*** *– which one best describes you?*

Married 🗆 Living with partner 🗆 Single 🗆 Divorced 🗆 Widowed 🗆

Other ................................ Number of children ................ Expected ................

**8) Finally, what was your age at last birthday:**…….......... years

***Thank respondent and give leaflet.***

***Officer initial*** ………… ***Date of screening****…………………*
